# In-Line Mach–Zehnder Interferometers Based on a Capillary Hollow-Core Fiber Using Vernier Effect for a Highly Sensitive Temperature Sensor

**DOI:** 10.3390/s21165471

**Published:** 2021-08-13

**Authors:** Sigifredo Marrujo-García, Iván Hernández-Romano, Daniel A. May-Arrioja, Vladimir P. Minkovich, Miguel Torres-Cisneros

**Affiliations:** 1 Electronics Department, DICIS, Universidad de Guanajuato, Carretera Salamanca-Valle de Santiago km 3.5 + 1.8, Salamanca 36885, Mexico; s.marrujogarcia@ugto.mx (S.M.-G.); torres.cisneros@ugto.mx (M.T.-C.); 2 CONACYT-Electronics Department, DICIS, Universidad de Guanajuato, Carretera Salamanca-Valle de Santiago km 3.5 + 1.8, Salamanca 36885, Mexico; 3 Fiber and Integrated Optics Laboratory (FIOLab), Centro de Investigaciones en Óptica A.C., Aguascalientes 20200, Mexico; darrioja@cio.mx; 4 Centro de Investigaciones en Óptica A.C., Calle Loma del Bosque 115, León 37150, Mexico; vladimir@cio.mx

**Keywords:** optical fiber sensors, capillary hollow-core fiber, Mach–Zehnder interferometer, Vernier effect

## Abstract

In this paper, we propose a highly sensitive temperature sensor based on two cascaded Mach–Zehnder interferometers (MZIs) that work using the Vernier effect. The all-fiber MZIs were assembled by splicing a segment of capillary hollow-core fiber (CHCF) between two sections of multimode fibers (MMFs). This cascaded configuration exhibits a temperature sensitivity of 1.964 nm/°C in a range from 10 to 70 °C, which is ~67.03 times higher than the sensitivity of the single MZI. Moreover, this device exhibits a high-temperature resolution of 0.0153 °C. A numerical analysis was carried out to estimate the devices’ temperature sensitivity and calculate the magnification of the sensitivity produced by the Vernier effect. The numerical results have an excellent agreement with the experimental results and provide a better insight into the working principle of the MZI devices. The sensor’s performance, small size, and easy fabrication make us believe that it is an attractive candidate for temperature measurement in biological applications.

## 1. Introduction

Fiber optic sensors (FOSs) have become an important technology and a wide field of research for the scientific community and industry. These devices offer outstanding features such as high sensitivity, a compact size, immunity to electromagnetic interference, and being lightweight. FOSs have been used to measure a vast number of physical variables such as refractive index (RI) [1], temperature [2], strain [3], pressure [4], and curvature [5], to mention a few. Among these parameters, temperature is an essential variable that needs to be monitored in almost all manufacturing processes and laboratory experiments. A large number of fiber optic temperature sensors have been assembled using tapered fiber [6], photonic crystal fiber [7], fiber Bragg grating (FBG) [8], long-period fiber grating (LPFG) [9], and multimode interference [10]. Moreover, fiber optic interferometers such as Mach–Zehnder [11], Fabry–Pérot (FP) [12], and Sagnac [13] have also been fabricated for temperature measurement. These devices take advantage of the thermo-optic and thermal-expansion effects of the silica to convert temperature changes into either wavelength shifts or power variations of the output spectrum. Although these fiber temperature sensors have shown promising results for high-temperature measurement, they have exhibited low sensitivity for temperatures lower than 80 °C. This low sensitivity occurs because the thermo-optic coefficient (TOC) (8.5 × 10^−6^/K) [14] and the thermal-expansion coefficient (TEC) (4.1 × 10^−7^/°C) [15] are low. One way to overcome this limitation is by using polymers since these materials exhibit a high TOC and TEC. It has been shown that the temperature sensitivity is increased by covering the sensing area of FOSs with a polymer. This technique has been implemented in sensors based on tapered fiber [16], FP cavity [17], surface plasmon resonance (SPR) [18], and FBG [19], to mention a few fiber structures, and this has improved the temperature sensitivity. Nevertheless, this also adds more complexity to the sensor fabrication process.

**Figure 1:**
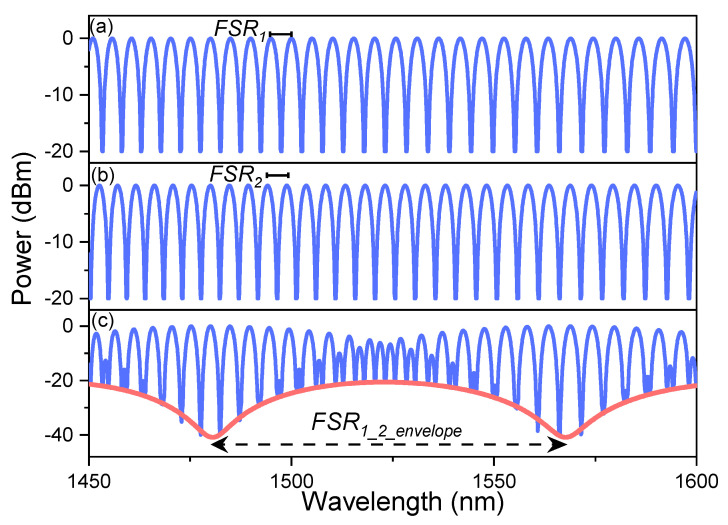
Interference patterns of (**a**) interferometer 1 and (**b**) interferometer 2, (**c**) the superposition of the two output signals (*FSR*_1_ ~ *FSR*_2_), using the Vernier effect in a series configuration.

Recently, the Vernier effect has been extensively studied as an effective method to increase the sensitivity of FOSs. The Vernier effect results from overlapping two interference patterns that have slightly different free spectral ranges (*FSR*), *FSR*_1_ ~ *FSR*_2_, where *FSR*_1_ and *FSR*_2_ are the *FSR* of the interferometer 1 and 2, respectively, see Figure 1a,b. Assuming that each interferometer undergoes a wavelength shift when a physical variable changes, then, one can determine the sensitivity of each device. In Vernier configuration, the superposition generates a signal with a large envelope (*FSR*_1_2_*envelope*_), see Figure 1c, showing a much larger wavelength shift (higher sensitivity) than that produced by a single interferometer. The overlap is achieved by connecting the interferometers, usually in a cascaded (series) configuration [20], but they can also be connected in parallel configuration [21,22]. The critical parameter in order to observe the Vernier effect is that the *FSR* of the two interference patterns is slightly different. When the *FSRs* are quite similar, the magnification (*M*) is larger, and higher sensitivity can be observed. For example, H. Liao et al. [20] assembled two MZIs to develop a temperature sensor based on a core offset technique, where the percentage of size difference (PSD) between the length of the devices was 10% (*M* = 8.7, the sensitivity increased from 0.04536 to 0.39736 nm/°C). Moreover, T. Paixão et al. [23] demonstrated a temperature sensor using two FPIs, fabricated with a femtosecond laser, where the PSD between the lengths of the cavities was 1% (*M* = 100, the sensitivity was increased up to 0.927 nm/°C). Besides, L. Y. Shao et al. [24] constructed two Sagnac interferometers by using polarization-maintaining fiber (PMF), and PSD between the lengths of PMFs, which formed part of each interferometer, was 13.17% (*M* = 9.15, experimental; *M* = 7.91, theoretical; the sensitivity increased from −1.4 to −13.36 nm/°C). It is well-known that the length of the fiber optic interferometer determines the FSR. Therefore, their fabrication process must be very precise to produce two interferometric devices with similar FSRs.

On the other hand, it has been reported that the combination of different fiber optic interferometers can also generate the Vernier effect. The combinations of interferometers that have been used for temperature measurement are Sagnac–FPI [25] and FPI–MZI [26]. By using two different interferometers, we could, in principle, eliminate the requirement of having quite similar fiber lengths on the interferometers since they can have very different length requirements as in the case of the Sagnac–FPI combination [25]. Nevertheless, the Vernier effect requires similar FSRs for both interferometers, and this is again related to meeting a specific fiber length. It is worth mentioning that, typically, interferometers requiring short fiber lengths exhibit larger *M* values, while interferometers with longer fiber lengths have lower *M* values. The latter is related to the more complicated control of longer fiber lengths.

In this paper, we propose, through numerical and experimental demonstration, a highly sensitive temperature sensor based on two cascaded MZIs that work using the Vernier effect. The all-fiber MZIs were assembled by sandwiching a segment of CHCF between two short sections of multimode fibers (MMFs). It was found that the temperature sensitivity of a single MZI was 29.3 pm/°C in a range from 10 to 70 °C, which was increased to a sensitivity of 1.964 nm/°C when the sensor was operated in a Vernier configuration (MZIs in series). The experimental results show that the temperature sensitivity of the single MZI was amplified ~67.03 times, and this significant amplification was possible thanks to the small length difference (~45 µm, PSD = 1.54%) between the CHCF segments of the MZIs. The enhanced performance of our series configuration is related to the optimization of the fiber structure and the high precision cutting system that we used during the fabrication process (allowing accurate control of the CHCF length of each MZI). Additionally, the temperature resolution of the two cascaded MZIs was 0.0153 °C, using the resolution of our interrogation system that was 30 pm. Finally, the advantages of having a high sensitivity, compact size, high resolution, and temperature range make the proposed two cascaded MZIs an appealing device for biological application. Moreover, we believe that this sensor is attractive for the emerging field of edge computing and sensing due to its low-weight and low-power consumption [27].

## 2. Working Principle

### 2.1. Numerical Simulation of a Single MZI and Its Temperature Sensitivity

**Figure 2:**
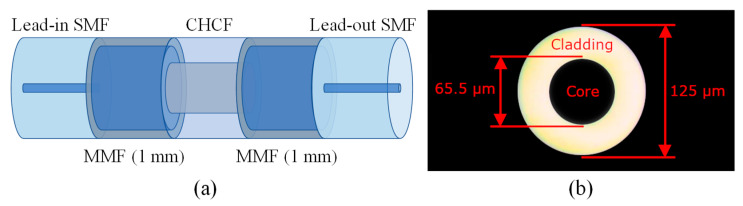
(**a**) Schematic drawing of an MZI based on one section of CHCF and two sections of MMFs (1 mm); (**b**) Cross-section of the CHCF.

A schematic drawing of the proposed single MZI structure is shown in Figure 2a. It consists of one section of CHCF that is sandwiched between two sections of MMFs. The inner and the outer diameters of the CHCF are 65.5 and 125 µm, respectively, see Figure 2b. Light from a broadband source is launched by lead-in single-mode fiber (SMF), and the output signal is sent to a detection system by lead-out SMF.

**Figure 3:**
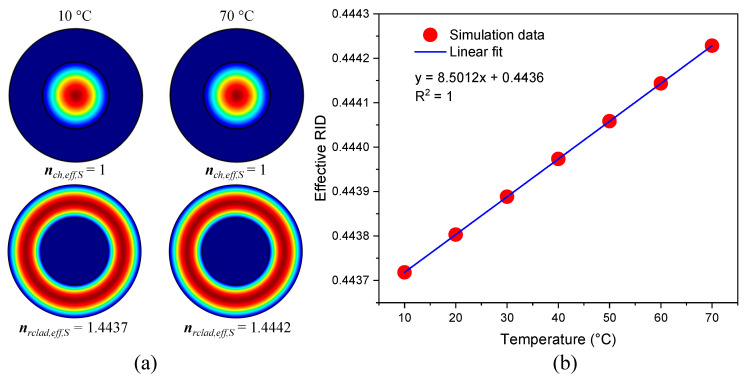
(**a**) Mode field distribution of the fundamental and cladding modes at two different temperatures (10 and 70 °C), (**b**) Simulation of the variation of the effective refractive index difference (ERID) at different temperatures.

A modal analysis regarding this MZI was carried out and it was found that two modes propagate in this structure. One of these was the fundamental mode that travels in the central hole of the CHCF, and the other was the cladding mode that propagates in the ring-cladding section, Figure 3a. By using the commercial software COMSOL, based on the finite-element method (FEM), the effective refractive index (ERI) of the fundamental mode ($n_{ch,eff,S}$) and ring-cladding mode ($n_{rclad,eff,S}$) is calculated. It is well-known that the RI of the silica is modified as the temperature changes, and the TOC ($\alpha_{TOC}$) is used to calculate the RI increment as the temperature is increased (${n=n}_{0\;}{+\;\alpha }_{TOC}\Delta T$, where $n_{0}$ is the RI given by the Sellmeier equation [28] at 20 °C, and ΔT is the temperature increment). Then, when the MZI undergoes temperature fluctuations, the RI of the silica is modified, causing the ERI of the mode that travels in the ring-cladding to also be changed. Since temperature fluctuations below 80 °C will hardly change the RI of the air, it is assumed that the ERI of the fundamental mode is the same during this simulation. The ERI of the two modes at two different temperatures is shown in Figure 3a. The ERI difference (ERID) ($\Delta n_{eff,S}{\;=\;n}_{ch,eff,S}\;{-\;n}_{rclad,eff,S}$) is calculated for different temperatures, and a linear relation between them is found out as shown in Figure 3b.

**Figure 4:**
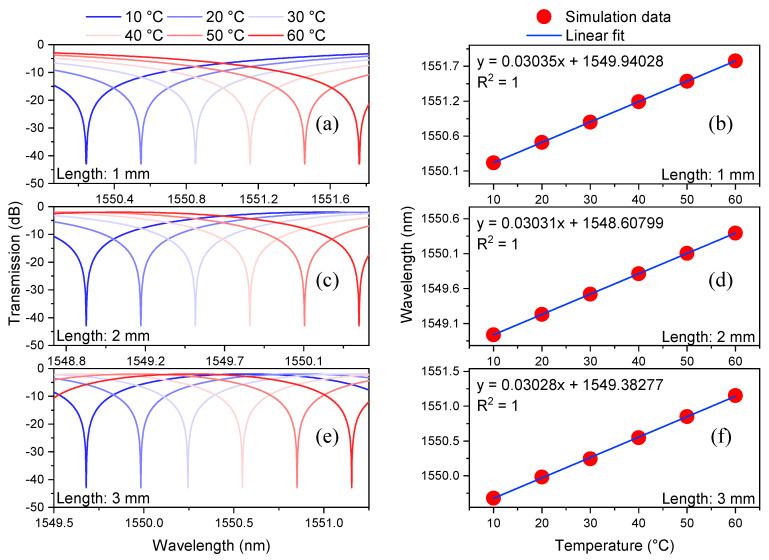
Numerical simulations of the transmission spectra of the MZIs at different temperatures using lengths ($L_{0S}$) of 1 mm (**a**), 2 mm (**c**), and 3 mm (**e**). The wavelength shift of one dip of the spectrum as a function of temperature using lengths ($L_{0S}$) of 1 mm (**b**), 2 mm (**d**), and 3 mm (**f**).

The equation that describes the transmission spectrum of this MZI is given by [5]

(1) $$I_{S}{\;=\;I}_{ch,S\;}{\;+\;I}_{rclad,S}\;+\;2\sqrt{I_{ch,S}I_{rclad,S}}cos\left\{\frac{{2\pi \Delta n}_{eff,s}}{\lambda }L_{s}\right\}\;$$

where $I_{ch,S}$ and $I_{rclad,S}$ are the intensities of the propagated beams in the central hole and in the ring-cladding, respectively. $L_{S}$ and λ are the length of the CHCF and the wavelength of the light, respectively. The value of $L_{S}$ increases as the temperature rises, and the TEC ($\alpha_{TEC}$) is used to calculate the length changes ($L_{S}{\;=\;L}_{0S}{\;+\;L}_{0S}\alpha_{TEC}\Delta T$). The different values of $L_{0S}$ used in this simulation start from 0.5 to 3 mm in steps of 0.5 mm. It is feasible to simulate the output spectrum of the MZI by using Equation (1), the ERID from Figure 3b, and the values of $L_{S}$ (see Figure 4). These simulations show the wavelength shift that the interference patterns undergo as the temperature changes from 10 to 60 °C in steps of 10 °C, for MZIs whose lengths ($L_{0S}$) are 1, 2, 3 mm, respectively, see Figure 4a,c,e. The linear response of the MZIs and their temperature sensitivity are shown in Figure 4b,d,f. Table 1 summarizes these results and includes the temperature sensitivity of devices whose lengths are 0.5, 1.5, and 2.5 mm. It is evident that the *FSR* of each sensor is decreasing as the length of the MZI is increasing. Since the temperature sensitivity of each device is quite similar, it is not possible to choose a sensor that exhibits better performance based on the results shown in Table 1. Instead of choosing a sensor based on its temperature sensitivity, we selected the MZI that exhibits the smallest fabrication error (in this work, the percentage error relative to the sensor’s length was used, it is defined as │measured value—actual value│/actual value). Achieving the smallest error is a key element that can increase or decrease the *M* factor of the Vernier effect, as will be shown in the next section.

### 2.2. Magnification of Vernier Effect

**Figure 5:**
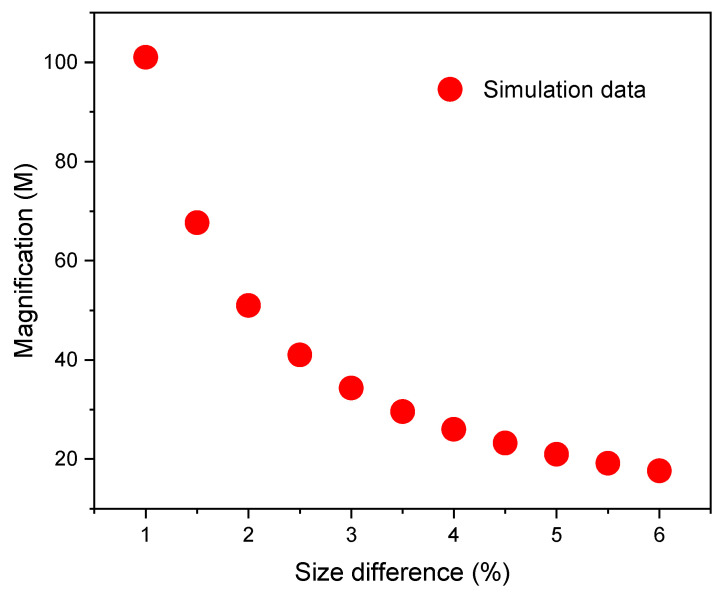
Variations of the magnification (*M*) as a function of the percentage of the size difference.

As it was mentioned before, the Vernier effect can be observed when two MZIs are set in series, with the first and the second sensors labeled as the sensing and the reference MZIs, respectively. The sensing MZI undergoes temperature variation, while the reference MZI is kept at a constant temperature. The superposition of their two interference patterns generates an envelope whose ${FSR}_{envelope}$ can be evaluated by [20]

(2) $${FSR}_{envelope\;}=\;\frac{{FSR}_{R}{FSR}_{S}}{\left|{FSR}_{R}\;-\;{FSR}_{S}\right|}\;$$

where ${FSR}_{S}$ and ${FSR}_{R}$ are the *FSR* of the sensing and reference MZIs, respectively. This equation is crucial because it determines the span that will be required to observe the wavelength shift. The magnification factor is another important value that is calculated with the help of the following expression [20]

(3) $$M\;=\;\frac{{FSR}_{R}}{\;\left|{FSR}_{R}-{FSR}_{S}\right|}\;=\;\frac{L_{0S}}{\left|L_{0S}-L_{0R}\right|}\;$$

where $L_{0S}$ and $L_{0R}$ are the lengths of the sensing and reference MZIs, respectively. Using the formula $FSR=\lambda^{2}/{\Delta n}_{eff}L$, it is possible to calculate the *FSR* of each MZI. It is evident that the ${FSR}_{S}$ and ${FSR}_{R}$ depend on the values of $L_{0S}$ and $L_{0R}$, respectively. Thus, the magnitude (*M*) becomes larger as the lengths of the two MZI get closer. Different values of *M* can be calculated using the percentage of size difference (PSD) between $L_{0S}$ and $L_{0R}$ without the need to specify any length. Additionally, it is possible to write $L_{0S}$ in terms of $L_{0R}$ as $L_{0S}=L_{0R}\;+\;L_{0R}\left(PSD\right)$, thus, using this equation and the second part of the Equation (3), the magnification can be expressed as M=(1+PSD)/PSD. It should be noted that if the PSD is less than 1%, the value of *M* is higher than 100, and if the PSD is more than 5%, the value of *M* is smaller than 20 (see Figure 5). For that reason, it is necessary to develop an accurate and reliable cutting system capable of cutting precise segments of CHCF since the fabrication error associated with this process determines the value of the magnification that can be achieved and is directly related with the ${FSR}_{envelope}$. Having a PSD equal to or smaller than 1% generates greater magnifications, but this can be a problem because a broader source and a wider wavelength span are required to track the envelope of the cascaded MZIs.

### 2.3. Numerical Simulation of Two Cascaded MZIs and Their Temperature Sensitivity

**Figure 6:**
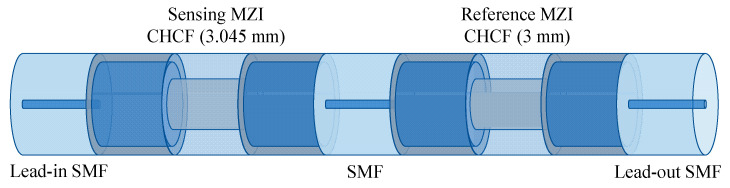
Schematic drawing of two cascaded MZI with different lengths.

This section presents a numerical analysis of two MZIs in a cascaded configuration, as shown in Figure 6. The sensing MZI undergoes temperature variation, while the reference MZI is kept at a constant temperature.

**Figure 7:**
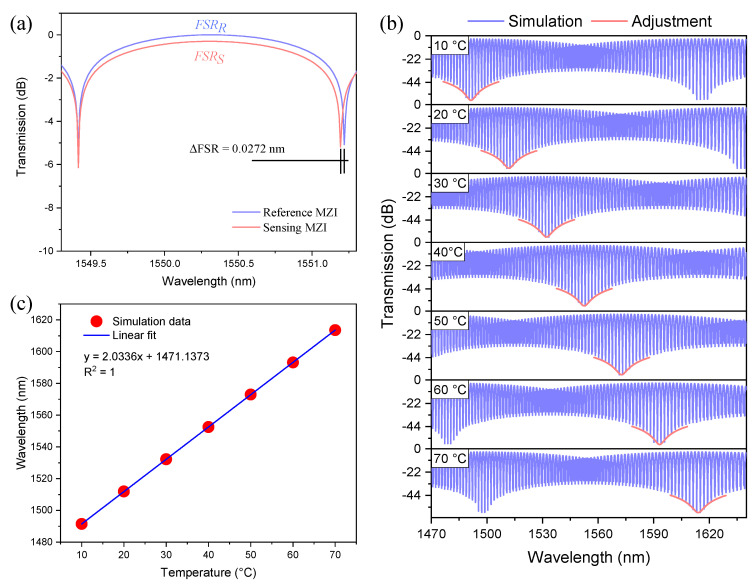
(**a**) Transmission spectra of the two MZIs with different lengths, (**b**) Transmission spectra of the two cascaded MZIs when the sensing MZI was at different temperatures, (**c**) Wavelength shift of the fitting enveloped dip as a function of temperature changes.

The transmission spectrum of each MZI is described by Equation (1), and these expressions can be rewritten to a similar form of the equations used in reference [20]. The interference spectra of the sensing and reference MZI are then given by

(4) $$I_{S}{\;=\;A}_{1}{\;+\;B}_{1}cos\left\{\frac{2\pi \Delta n_{eff,S}}{\lambda }L_{S}\right\}\;$$

(5) $$I_{R}{\;=\;A}_{2\;}{+\;B}_{2}cos\left\{\frac{2\pi \Delta n_{eff,R}}{\lambda }L_{R}\right\}\;$$

where $A_{1}{\;=\;I}_{ch,S}{\;+\;I}_{rclad,S}$, $A_{2}{\;=\;I}_{ch,R}{\;+\;I}_{rclad,R}$, $B_{1\;}=\;2\sqrt{I_{ch,S}I_{rclad,S}}$, and $B_{2\;}=\;2\sqrt{I_{ch,R}I_{rclad,R}}$. Here, we are assuming that the length of the reference MZI ($L_{0R}=3\;\mathrm{mm}$) is smaller than the length ($L_{0S\;}=3.045\;\mathrm{mm}$) of the sensing MZI, i.e., $L_{0R}$ is 1.5% smaller than $L_{0S}$. These lengths were chosen because, as will be shown in the experimental section, the lowest fabrication errors are obtained for CHCF segments of approximately 3 mm. Therefore, each MZI generates a specific *FSR,* which can be observed in Figure 7a. The transmission spectrum of these two cascaded MZIs are calculated by multiplying Equations (3) and (4), and the result can be written as [20]

(6) $$\begin{array}{c} {I\;=\;A}_{1}A_{2}{+A}_{2}B_{1}cos\left\{\frac{2\pi \Delta n_{eff,S}}{\lambda }L_{S}\right\}{+A}_{1}B_{2}cos\left\{\frac{2\pi \Delta n_{eff,R}}{\lambda }L_{R}\right\}+\\ \frac{1}{2}B_{1}B_{2}cos\left\{\frac{2\pi }{\lambda }\left[\Delta n_{eff,S}L_{S}+\Delta n_{eff,R}L_{R}\right]\right\}+\frac{1}{2}B_{1}B_{2}cos\left\{\frac{2\pi }{\lambda }\left[\Delta n_{eff,S}L_{S}-\Delta n_{eff,R}L_{R}\right]\right\} \end{array}$$

The values of $\Delta n_{eff,S}$ and $L_{S}$ change as the temperature varies from 10 to 70 °C, while the values of $\Delta n_{eff,R}$ and $L_{R}$ are calculated at a fixed temperature of 30 °C. The transmitted intensity given by Equation (6) as a function of wavelength for different temperature values is shown in Figure 7b. The transmission spectra show a high-frequency interference pattern modulated by a low-frequency envelope (*FSR_envelope_* = 117.9 nm). For sensing applications, monitoring the wavelength shift of the low-frequency envelope is carried out by fitting one of its dips. As shown in Figure 7b, the dip of the envelope experiences a redshift as the temperature is increased. It is worth mentioning that the temperature sensitivity of the two cascaded MZIs is 2.0336 nm/°C, which can be estimated from Figure 7c. Comparing the temperature sensitivity of the sensing MZI with the two cascaded MZIs (M=(2.0336 nm/°C)/(0.03028 nm/°C)), it is quite easy to realize that the temperature sensitivity of the two cascaded MZIs is ~67.16 times higher than the sensitivity of the single MZI.

## 3. Fabrication and Experimental Results

### 3.1. Fabrication Process of the MZI

**Figure 8:**
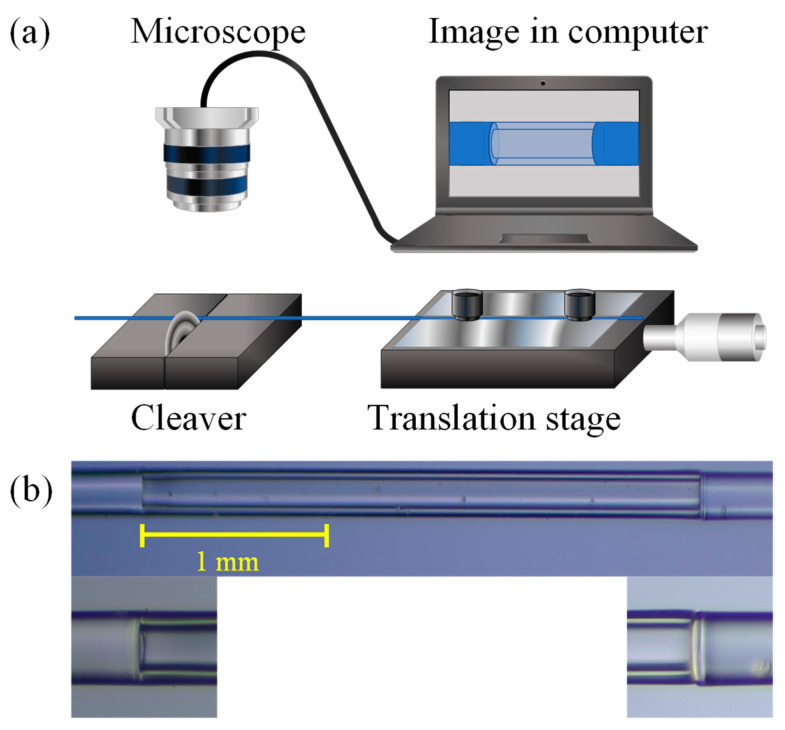
(**a**) Experimental setup for cutting a segment of MMF or CHCF accurately. (**b**) Lateral view of the fabricated device and the splicing section between MMF and CHCF.

As previously explained, the fabrication of two similar MZIs is vital in this work since connecting both in Vernier configuration generates large values of *M*. The fabrication process used in this research depends on an experimental setup designed to cleave the CHCF lengths accurately. This setup consists of a translation stage where a segment of SMF is set in a v-groove and held with the help of two magnets; meanwhile, the part of the optical fiber that needs to be cut rests on a fiber cleaver (see Figure 8a). Then, the SMF tip is set in a fusion splicer to splice it, either with an MMF or a CHCF. After splicing, the fiber is put back into the cleaver where the splicing section is above the blade, corresponding to the zero position. Afterwards, the micrometer screw of the translation stage moves to a specific length, and the fiber (MMF or CHCF) is cut with high precision using the cleaver. All of these steps are carried out without removing the magnets that attach the SMF to the translation stage. Additionally, a microscope connected to a computer is used to observe the initial position of the optical fiber to have better control of the cutting process.

Before the MZI fabrication process is described, the features of the optical fibers used in this work are shown in Table 2.

**Figure 9:**
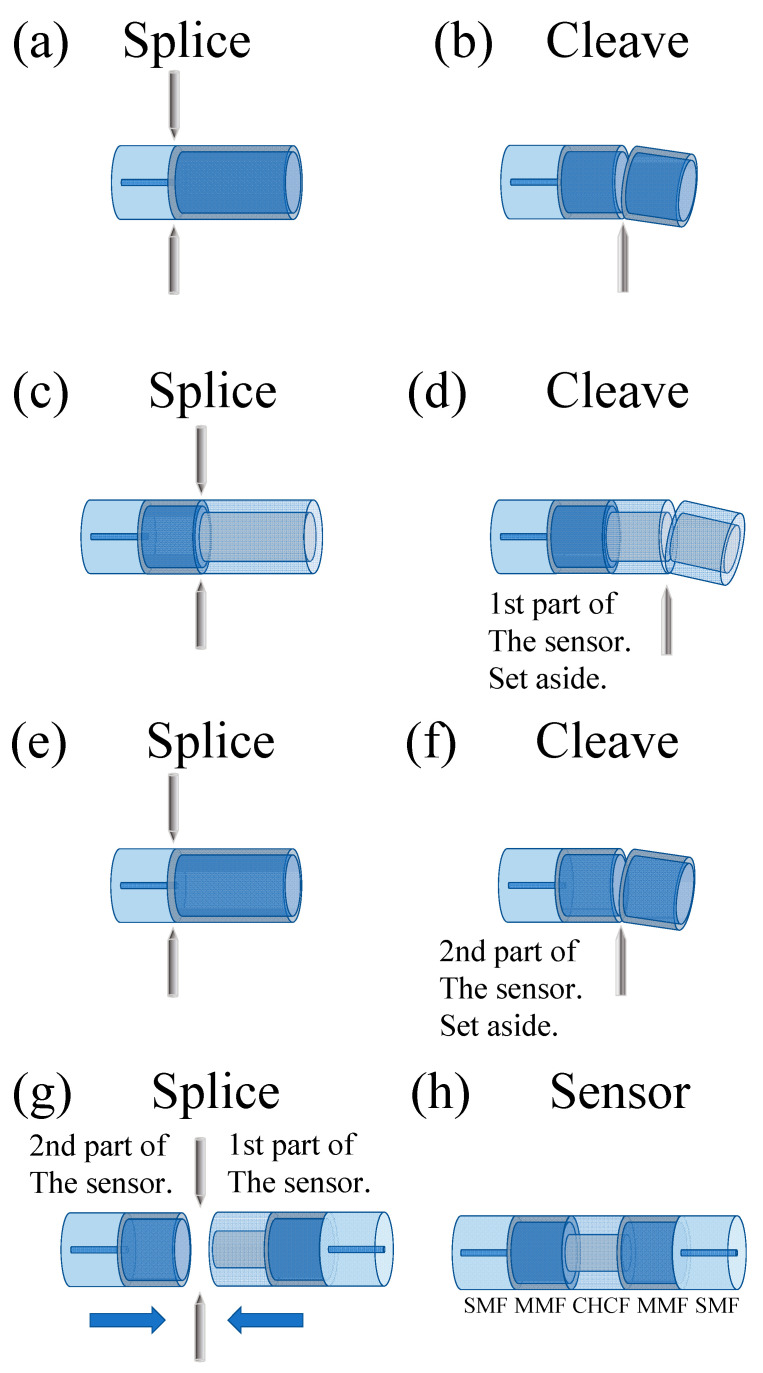
The fabrication process of the MZI: Fabrication of the first part (**a**–**d**), Fabrication of the second part (**e**) and (**f**), Assembly of the MZI (**g**) and (**h**).

The fabrication process of this MZI can be separated into two parts, as shown in Figure 9. The experimental setup described in Figure 8 was used to construct this device. The first part of this process consisted of splicing an SMF to an MMF, and then a section of MMF (1 mm) was cleaved. This MMF section works as a beam splitter to the interferometer. After that, the MMF was spliced to a CHCF, and then a section of CHCF was cleaved to a specific length (we used lengths from 0.5 to 3 mm in steps of 0.5 mm), see Figure 9a–d. The second part comprised constructing another beam splitter by splicing and cleaving a section of MMF to an SMF, as shown in Figure 9e,f. Finally, to complete the fabrication process of the MZI, it was necessary to splice these two parts, as shown in Figure 9g,h. Figure 8b shows a lateral view of the fabricated devices as well as the splicing section between MMF and CHCF.

We should highlight that a special splicing program was developed to minimize splice losses and avoid deformation of the CHCF that could modify the amplitude of the cladding mode (causing lower contrast in the interference pattern). A Fitel fusion splicer (model s179) was used in this fabrication process, and the parameters of the program are shown in Table 3.

**Figure 10:**
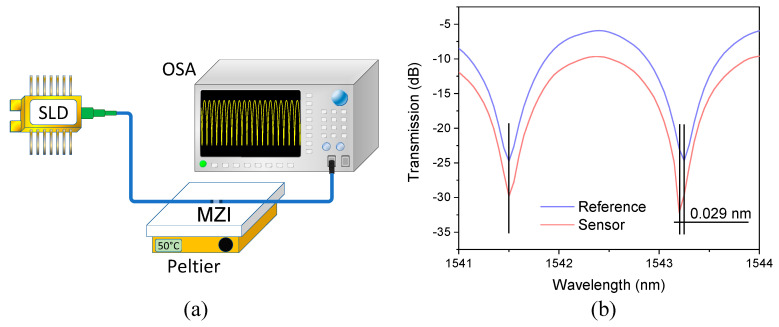
(**a**) Experimental setup for testing the transmission of the MZIs, (**b**) The transmission spectra of the sensing and reference MZIs at a reference temperature of 30 °C.

At this point, additional tests were carried out to verify the fabrication error of the MZIs that were implemented. This means that we were interested in determining the percentage error generated when we cleaved a specific length of CHCF. It should be noted that it is possible to calculate the length of an MZI using $L\;=\;\lambda^{2}/{\Delta n}_{eff}\left(FSR\right)$), for that purpose, an experimental setup that allowed us to investigate the *FSR* using the transmission spectrum of each MZI was assembled, as shown in Figure 10a. The experimental setup consisted of a superluminescent diode (SLD) centered at 1550 nm with a spectral bandwidth of 160 nm. Light from the SLD was sent to an MZI using a lead-in SMF, and the transmitted signal of the device was delivered to an optical spectrum analyzer (OSA) (Anritsu MS9740A) with a lead-out SMF. Before making any measurement, each sensor was set on a Peltier (Echotherm IC20 Digital, Torrey Pines) at a constant temperature (30 °C). This reference temperature was selected to redshift a valley of the Vernier envelope to the left of the available SLD spectrum (as in the case presented in Figure 7, when the sensing MZI was at 10 °C). Once this value was determined, five MZIs of each length were fabricated to estimate each length’s fabrication error (percentage error), as shown in Table 4. Column 2 shows the fabrication length errors and the average of these values is ~50 µm, meaning that the error is almost the same for all the lengths that were cut. Column 3 indicates the percentage errors for all lengths; as was mentioned before, this relates the desired length that needs to be cut with the error in the cutting process. Therefore, it can be observed that the percentage errors decrease as the length of the CHCF increases since the fabrication length error is almost the same for all the lengths. We believe that the errors in the fabrication process come from the total allowable error within the items used; to mention some of them: translation stage (minimum achievable incremental movement, 10 µm, angular deviation, <150 µrad, backlash, <3 µm); cleaver (cleaving angle, <3°); how fiber is fixed; alignment of the fiber splicing to the cleaver blade (zero position). Besides, it is worth noticing that the magnification factor (column 5) increases as the length of the CHCF increases. We should highlight that MZIs with CHCF lengths larger than 3 mm were fabricated, but their fringe contrast was less than 10 dB. Therefore, we decided to select lengths close to 3 mm to fabricate the MZIs since this length has the lower fabrication error. Using this device, we could generate a considerably larger value of *M*. Two MZIs were chosen, and their transmission spectra are depicted in Figure 10b. We can observe that the *FSR* of each interference pattern is slightly different (${FSR}_{R}\;=\;1.859\;\mathrm{nm}$ and ${FSR}_{S}=1.830\;\mathrm{nm}$), and the *FSR* difference between these spectra is 0.029 nm. Using the value of each *FSR*, it was found that the CHCF lengths of the reference and the sensing sensors were $L_{0R}=2912.656\;\mu m$ and $L_{0S}=2957.576\;\mu m$, respectively. The length difference of the CHCF corresponded to 1.54%, which is only 0.04% higher than that used in the simulation.

### 3.2. Experimental Results of a Single MZI as a Temperature Sensor

**Figure 11:**
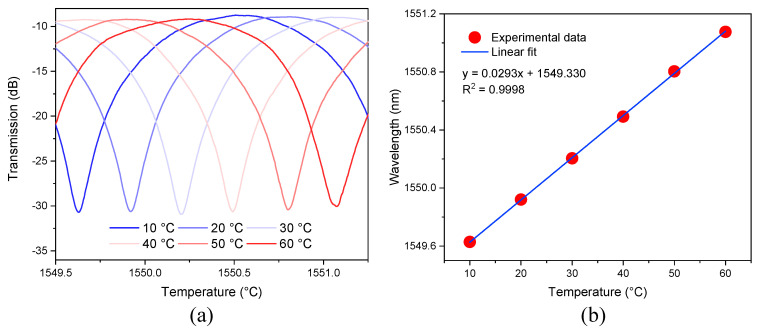
(**a**) Experimental spectra of the sensing MZI at different temperatures, (**b**) Wavelength shift of one dip of the spectrum as a function of temperature.

The primary objective of our work is to demonstrate that the temperature sensitivity of the sensing MZI is improved by assembling it in a cascaded configuration with a reference MZI via the Vernier effect. Therefore, to validate our theory, the first step was to characterize the sensing MZI at different temperatures by using the experimental setup shown in Figure 10a. Light from the SLD was sent to the sensing MZI using a lead-in SMF, and the output transmission of the device was delivered to an OSA by a lead-out SMF. The sensing MZI was set on a Peltier to increase the temperature from 10 to 60 °C in steps of 10 °C. The spectral response of the sensing MZI at different temperatures is plotted in Figure 11a, where a redshift of the transmitted spectrum can be observed by tracking one of the dips in the spectrum. Figure 11b shows the wavelength shift of the dip as the temperature increases, and temperature sensitivity of 29.3 pm/°C was estimated. We should highlight that the difference between simulated and experimental temperature sensitivities is only 3.24%, which means that our numerical analysis agrees reasonably well with the experimental results.

### 3.3. Experimental Results of Two Cascaded MZIs and the Vernier Effect

**Figure 12:**
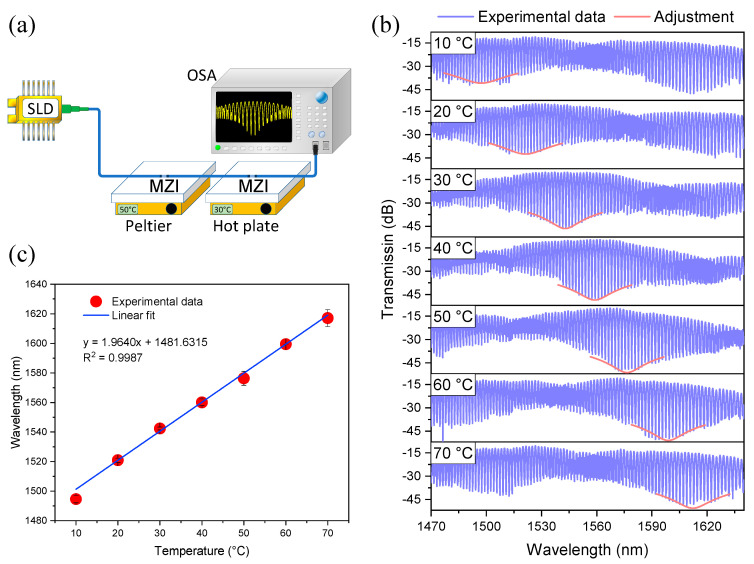
(**a**) Experimental setup for temperature measurement based on Vernier effect. (**b**) Experimental spectra of the two MZIs when the sensing MZI was at different temperatures, (**c**) Wavelength shift of one dip of the lower envelope as a function of temperature.

The temperature response of the two MZIs connected in a cascaded configuration was tested by using the experimental setup shown in Figure 12a. This experimental setup works in the same way the previous setup but, in this case, the sensing MZI was set on a Peltier to increase the temperature from 10 to 70 °C in steps of 10 °C; meanwhile, the reference MZI was set on a hot plate at the fixed temperature of 30 °C (these sensors were separated by 1 m of SMF). The spectral response of the two cascaded MZIs under these experimental conditions is shown in Figure 12b. We observe that the spectral response of the concatenated MZIs generated an interference pattern modulated with a low-frequency envelope ($FSR_{envelope}=113.3\;\mathrm{nm}$). Comparing the simulated and the experimental value of the $FSR_{envelope}$, one can observe that the difference between them is 4.6 nm (3.9%). One dip of the lower envelope was monitored to measure its wavelength shift to know the temperature sensitivity of the two cascaded MZIs. It should be mentioned that the dip distortion at the wavelength span edges is related to the low power of the SLD. This experiment was repeated three times, and the mean values are plotted in Figure 12c, where the maximum error associated with these measurements was 2.48%. The temperature sensitivity of the two cascaded MZIs was 1.964 nm/° C. It is worth noting that the difference between simulated and experimental temperature sensitivity is 3.42%, which again validates our numerical analysis since it provides an excellent agreement with the experimental results. By taking advantage of the Vernier effect, in the second experimental setup, we observed that the temperature sensitivity of the two cascaded MZIs is ~67.03 times higher than the sensitivity of the sensing MZI by itself. The difference between simulated and experimental magnification factors is 0.2%.

### 3.4. Discussion

It has been shown numerically and experimentally that the temperature sensitivity of an all-fiber MZI can be increased by connecting two cascaded MZIs, with a small difference between their *FSRs*, and the enhanced sensitivity is due to the Vernier effect. The predicted temperature sensitivity by our proposed model exhibits an excellent agreement with the experimental results in both cases (one MZI and the two cascaded MZIs).

During the design of this experiment based on the Vernier effect, we focused our attention on three parameters: temperature sensitivity, temperature range, and the magnification factor. The enhancement in the temperature sensitivity is related to the magnification factor; likewise, the magnification factor determines the span required to analyze the optical spectrum, and it also affects the temperature sensing range. Therefore, those three parameters and the relation between them need to be optimized to enhance the sensitivity of sensors based on the Vernier effect.

At this point, it is necessary to do a review of all-fiber optic sensors based on the Vernier effect that has been published recently. This review allows us to compare our sensor with different Vernier configurations, see Table 5. It should be noted that sensors 3 and 6 were constructed by a combination of interferometers Sagnac/Fabry–Pérot and Fabry–Pérot/Mach–Zehnder, respectively. That is a common technique used to separate the sensing and the reference devices, with the purpose being that the sensing device undergoes different temperatures by itself. The problem with this configuration is the cumbersome process of fabricating interferometers (Sagnac or an MZI) with an FSR similar to the FSR of the FPI. Sensors 1, 5, and 8 are based on FPIs where the sensing and reference interferometers are in the fiber tip. Although those configurations offer the advantages of compact size and easy handling, it is not possible to separate the sensing and the reference sensors, causing both FPIs to experiment the same temperature. The sensors 2, 4, 7, 9, and our proposed structure are constructed using the same fiber interferometers, Sagnac, MZ, FP, MZ, and MZ, respectively. Since these sensors are based on interferometers with similar characteristics (such as length), the fabrication error of these devices should be small to generate two interferometers with almost equal *FSR*.

Regarding the temperature sensitivity of the sensing devices, one can observe that sensors 2, 3, 4, and 9 have higher sensitivity than ours. Our proposed structure has higher temperature sensitivity in Vernier configuration than sensors 1, 4, 5, 6, 7, 8, and 9; only sensors 2 and 3 have higher temperature sensitivity. It should be pointed out that sensors 2 and 3 are based on a Sagnac interferometer that requires a few meters of fiber to be constructed, whereas our proposed MZI has 5 mm of length. Due to the small size of our sensor as well as its temperature sensing range, it can be used for monitoring biological and chemical solutions and small devices as microchannels. Our proposed sensor has a magnification factor higher than sensors 2, 3, 4, and 9, and it has a similar factor to 5. One advantage of our sensor is that the sensing and reference MZIs can be separated. This lets the sensing MZI undergo different temperatures while the reference MZI is at a constant temperature. The above comparison makes us believe that our sensor has equal or better features than the state-of-the-art sensors based on the Vernier effect.

## 4. Conclusions

In summary, we propose and demonstrate a highly sensitive temperature sensor based on two cascaded all-fiber MZIs that exploit the Vernier effect. Using this configuration, it was possible to obtain a temperature sensitivity of 1.964 nm/°C in a range from 10 to 70 °C, which is ~67.03 times higher than the sensitivity of the single MZI. A numerical analysis verified these experimental results. Besides, this device exhibits a high-temperature resolution of 0.0153 °C. All of these outstanding features and the easy fabrication of the MZI make this proposed sensor an excellent candidate for biological applications requiring high resolution and sensitivity.

## Figures and Tables

**Table 1:** Simulation of MZIs using different lengths.

Length of the CHCF (mm)	FSR (nm)	Response (nm/°C)
0.5	10.829	0.03029
1.0	5.415	0.03035
1.5	3.610	0.03029
2.0	2.707	0.03031
2.5	2.166	0.03028
3.0	1.805	0.03028

**Table 2:** Parameter of the optical fibers.

Type	SMF	MMF	CHCF
Cladding diameter	125 µm	125 µm	125 µm
Core diameter	9 µm	105 µm	65.5 µm
Cladding index	1.443	1.443	1.443
Core index	1.452	1.462	1
Length	50 cm	1 mm	from 0.5 to 3 mm

**Table 3:** Parameters of the splicing program (Fitel, model s179).

Splicing Parameter Name	Parameter Values
1st Arc start power ^1^	10
1st Arc end power ^1^	40
1st Arc duration (ms)	1500
Cleaning offset/arc ^1^	10
Cleaning duration (ms)	100
Pre Arc duration (ms)	160
Gap (µm)	22
Z push length (µm)	15
Z pull start time (ms)	500
Z pull length (µm)	10
Alignment type	Cladding
Axis offset (µm)	0

^1^ The software in the splicer does not mention any unit regarding the arc power. We can only choose values from 0 to 255.

**Table 4:** Fabrication error of each length.

Length (mm)	Fabrication Length Error (mm) ^1^	Fabrication Error ^2^ (%)	FSR Envelope Based on Fabrication Error (nm)	Magnification Based on Fabrication Error (M)
0.5	0.0471	9.432	106.895	11.602
1.0	0.0354	3.545	157.263	29.209
1.5	0.0557	3.713	96.789	26.932
2.0	0.0625	3.125	85.115	33.000
2.5	0.0472	1.888	125.971	53.966
3.0	0.0470	1.569	107.654	64.735
Average:	0.0500	−	−	−

^1^ Absolute error of the fabrication process. ^2^ Percentage error relative to the sensor’s length.

**Table 5:** Different all-fiber temperature sensors based on the Vernier effect.

	Fiber Structure		Temperature Sensitivity			
	Sensing Device	Reference Device	Sensing Device (nm/°C)	Vernier Configuration (nm/°C)	ΔT (°C)	Magnification Factor (M)
1	SMF as an FPI (2015) [29]	SHCF as FPI	0.008	1.019	250–300	127.375
2	Sagnac interferometer (2015) [24]	Sagnac interferometer	−1.46	−13.36	0–10	9.15
3	Sagnac interferometer (2017) [25]	CHCF as FPI	−1.4	−29.0	42–44	20.7
4	MZI based on core offset (2017) [20]	MZI based core offset	0.04536	0.39736	10–75	8.7
5	Small-size FPI (using ion beam) (2019) [30]	FPI-air cavity	0.0097 from reference	−0.654	30–120	67.42
6	CHCF–FPI (2019) [26]	MZI using 3dB couplers	0.0012	−0.1072	30–80	89
7	FPI by using a femtosecond laser (2019) [23]	FPI by using a femtosecond Laser	Not mentioned	0.927	30–60	100
8	Hollow microsphere as FPI (2020) [31]	MMF as FPI	0.0072	−0.650	20–100	90.27
9	MZI based on CHCF [22]	MZI based on CHCF	0.03015	0.5285	0–100	17.5
10	Proposed sensor CHCF-MZI	CHCF-MZI	0.0291	1.964	10–70	67.03

## Data Availability

Not applicable.

## References

[1] Wang Q., Kong L., Dang Y., Xia F., Zhang Y., Zhao Y., Li J. (2016). High sensitivity refractive index sensor based on splicing points tapered SMF-PCF-SMF structure Mach-Zehnder mode interferometer. Sens. Actuators B Chem..

[2] Zhang Z., Liao C., Tang J., Wang Y., Bai Z., Li Z., Guo K., Deng M., Cao S., Wang Y. (2017). Hollow-core-fiber-based interferometer for high-temperature measurements. IEEE Photon. J..

[3] Zuo G., Li W., Yang Z., Li S., Qi R., Huang Y., Xia L. (2020). Double Phase Matching in MZI With Antiresonant Effect for Optical Fiber Sensor Application. J. Light. Technol..

[4] Gao H., Jiang Y., Zhang L., Cui Y., Jiang Y., Jia J., Jiang L. (2019). Antiresonant mechanism based self-temperature-calibrated fiber optic Fabry–Perot gas pressure sensors. Opt. Express.

[5] Choi H.Y., Kim M.J., Lee B.H. (2007). All-fiber Mach-Zehnder type interferometers formed in photonic crystal fiber. Opt. Express.

[6] Raji Y.M., Lin H.S., Ibrahim S.A., Mokhtar M.R., Yusoff Z. (2016). Intensity-modulated abrupt tapered fiber Mach-Zehnder interferometer for the simultaneous sensing of temperature and curvature. Opt. Laser Technol..

[7] Wu D., Zhao Y., Li J. (2015). PCF taper-based Mach–Zehnder interferometer for refractive index sensing in a PDMS detection cell. Sens. Actuators B Chem..

[8] Massaroni C., Caponero M.A., D’Amato R., Lo Presti D., Schena E. (2017). Fiber Bragg grating measuring system for simultaneous monitoring of temperature and humidity in mechanical ventilation. Sensors.

[9] Sun C., Han Z., Zhang S., Duan S., Jin X., Chen X., Yao C., Geng T., Zhang Z., Qu Z. (2019). A micro MMF layer embedded in LPFG for simultaneous measurement of curvature and temperature. Opt. Fiber Technol..

[10] Zhao Y., Cai L., Li X.G. (2017). In-fiber modal interferometer for simultaneous measurement of curvature and temperature based on hollow core fiber. Opt. Laser Technol..

[11] Gong H., Xiong M., Qian Z., Zhao C.L., Dong X. (2015). Simultaneous measurement of curvature and temperature based on Mach–Zehnder interferometer comprising core-offset and spherical-shape structures. IEEE Photon. J..

[12] Liu Y., Zhang T., Wang Y., Yang D., Liu X., Fu H., Jia Z. (2018). Simultaneous measurement of gas pressure and temperature with integrated optical fiber FPI sensor based on in-fiber micro-cavity and fiber-tip. Opt. Fiber Technol..

[13] Bai Y., Miao Y., Zhang H., Yao J. (2020). Simultaneous measurement of temperature and relative humidity based on a microfiber Sagnac loop and MoS 2. J. Light. Technol..

[14] Komma J., Schwarz C., Hofmann G., Heinert D., Nawrodt R. (2012). Thermo-optic coefficient of silicon at 1550 nm and cryogenic temperatures. Appl. Phys. Lett..

[15] Li X., Lin S., Liang J., Zhang Y., Oigawa H., Ueda T. (2011). Fiber-optic temperature sensor based on difference of thermal expansion coefficient between fused silica and metallic materials. IEEE Photon. J..

[16] Hernández-Romano I., Monzón-Hernández D., Moreno-Hernández C., Moreno-Hernandez D., Villatoro J. (2015). Highly sensitive temperature sensor based on a polymer-coated microfiber interferometer. IEEE Photon. Technol. Lett..

[17] Hernández-Romano I., Cruz-Garcia M.A., Moreno-Hernández C., Monzón-Hernández D., López-Figueroa E.O., Paredes-Gallardo O.E., Villatoro J. (2016). Optical fiber temperature sensor based on a microcavity with polymer overlay. Opt. Express.

[18] Velázquez-González J.S., Monzón-Hernández D., Moreno-Hernández D., Martínez-Piñón F., Hernández-Romano I. (2017). Simultaneous measurement of refractive index and temperature using a SPR-based fiber optic sensor. Sens. Actuators B Chem..

[19] Liu Y., Guo Z., Zhang Y., Chiang K.S., Dong X. (2000). Simultaneous pressure and temperature measurement with polymer-coated fibre Bragg grating. Electron. Lett..

[20] Liao H., Lu P., Fu X., Jiang X., Ni W., Liu D., Zhang J. (2017). Sensitivity amplification of fiber-optic in-line Mach–Zehnder Interferometer sensors with modified Vernier-effect. Opt. Express.

[21] Nan T., Liu B., Wu Y., Wang J., Mao Y., Zhao L., Wang J. (2019). Ultrasensitive strain sensor based on Vernier-effect improved parallel structured fiber-optic Fabry-Perot interferometer. Opt. Express.

[22] Wang Z., Huang L., Liu C., Wang H., Sun S., Yang D. (2019). Sensitivity-Enhanced Fiber Temperature Sensor Based on Vernier Effect and Dual In-Line Mach–Zehnder Interferometers. IEEE Sens. J..

[23] Paixão T., Araújo F., Antunes P. (2019). Highly sensitive fiber optic temperature and strain sensor based on an intrinsic Fabry–Perot interferometer fabricated by a femtosecond laser. Opt. Lett..

[24] Shao L.Y., Luo Y., Zhang Z., Zou X., Luo B., Pan W., Yan L. (2015). Sensitivity-enhanced temperature sensor with cascaded fiber optic Sagnac interferometers based on Vernier-effect. Opt. Commun..

[25] Yang Y., Wang Y., Zhao Y., Jiang J., He X., Yang W., Li L. (2017). Sensitivity-enhanced temperature sensor by hybrid cascaded configuration of a Sagnac loop and a FP cavity. Opt. Express.

[26] Ying Y., Zhao C., Gong H., Shang S., Hou L. (2019). Demodulation method of Fabry-Perot sensor by cascading a traditional Mach-Zehnder interferometer. Opt. Laser Technol..

[27] Passian A., Imam N. (2019). Nanosystems, edge computing, and the next generation computing systems. Sensors.

[28] Malitson I.H. (1965). Interspecimen comparison of the refractive index of fused silica. Josa.

[29] Zhang P., Tang M., Gao F., Zhu B., Zhao Z., Duan L., Fu S., Ouyang J., Wei H., Shum P.P. (2015). Simplified hollow-core fiber-based Fabry–Perot interferometer with modified Vernier effect for highly sensitive high-temperature measurement. IEEE Photon. J..

[30] Gomes A.D., Becker M., Dellith J., Zibaii M.I., Latifi H., Rothhardt M., Frazão O. (2015). Multimode Fabry–Perot interferometer probe based on Vernier effect for enhanced temperature sensing. Sensors.

[31] Gomes A.D., Ferreira M.S., Bierlich J., Kobelke J., Rothhardt M., Bartelt H., Frazão O. (2020). Hollow microsphere combined with optical harmonic Vernier effect for strain and temperature discrimination. Opt. Laser Technol..

